# Role for IL-1 Family Cytokines in Fungal Infections

**DOI:** 10.3389/fmicb.2021.633047

**Published:** 2021-02-10

**Authors:** James S. Griffiths, Giorgio Camilli, Natalia K. Kotowicz, Jemima Ho, Jonathan P. Richardson, Julian R. Naglik

**Affiliations:** Centre for Host-Microbiome Interactions, Faculty of Dentistry, Oral and Craniofacial Sciences, King’s College London, London, United Kingdom

**Keywords:** fungi, fungal immunology, IL-1, *Candida*, *Aspergillus*

## Abstract

Fungal pathogens kill approximately 1.5 million individuals per year and represent a severe disease burden worldwide. It is estimated over 150 million people have serious fungal disease such as recurrent mucosal infections or life-threatening systemic infections. Disease can ensue from commensal fungi or new infection and involves different fungal morphologies and the expression of virulence factors. Therefore, anti-fungal immunity is complex and requires coordination between multiple facets of the immune system. IL-1 family cytokines are associated with acute and chronic inflammation and are essential for the innate response to infection. Recent research indicates IL-1 cytokines play a key role mediating immunity against different fungal infections. During mucosal disease, IL-1R and IL-36R are required for neutrophil recruitment and protective Th17 responses, but function through different mechanisms. During systemic disease, IL-18 drives protective Th1 responses, while IL-33 promotes Th2 and suppresses Th1 immunity. The IL-1 family represents an attractive anti-fungal immunotherapy target. There is a need for novel anti-fungal therapeutics, as current therapies are ineffective, toxic and encounter resistance, and no anti-fungal vaccine exists. Furthering our understanding of the IL-1 family cytokines and their complex role during fungal infection may aid the development of novel therapies. As such, this review will discuss the role for IL-1 family cytokines in fungal infections.

## Introduction

Fungal pathogens represent an increasingly severe disease burden and are responsible for ∼1.5 million deaths per year. Patients who are immunocompromised, have undergone invasive clinical procedures or suffered trauma are particularly susceptible to fungal infection. Fungi can be frequently encountered, such as *Aspergillus* through inhalation or *Candida*, which colonizes mucosal barriers (Brown et al., 2012). Anti-fungal responses must strike a careful balance to provide protection and maintain homeostasis. Regularly encountered fungal pathogens must be cleared with minimal effect on the host, while commensal fungi must be maintained without reducing barrier integrity. The majority of serious fungal disease arises from poorly cleared infection or disrupted barrier integrity (Rautemaa-Richardson and Richardson, 2017). Here, the IL-1 family play a crucial role mediating both barrier and systemic anti-fungal immunity. As such, modulating IL-1 family cytokines to enhance anti-fungal immunity may provide valuable therapeutic strategies that overcome current therapeutic inadequacies.

**FIGURE 1:**
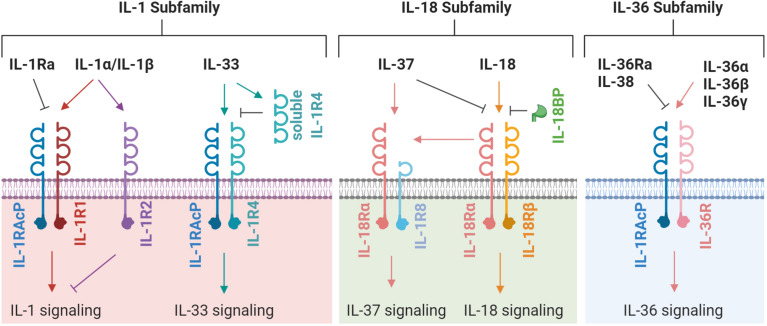
A schematic representation of IL-1 family receptors, co-receptors, and ligands. Each ligand, receptor and co-receptor are separated into their appropriate IL-1 subfamily. An arrow indicates the induction of signaling; a flat line indicates the blocking of signaling. The latest nomenclature is used, for previous/alternative nomenclature see Table 1.

The IL-1 family possess numerous potent biological activities and mediate a wide range of immunological responses (Garlanda et al., 2013). IL-1 was identified in the 1980’s but had been investigated for many years under various aliases. The discovery of IL-1 was initially met with skepticism that a molecule at such low concentration could have potent, systemic effect. Since then, our understanding of the IL-1 family has grown and now comprises four sub-families containing eleven signaling members, five primary receptors and six co-receptors (Figure 1). Of the eleven signaling members, seven are pro-inflammatory and four are anti-inflammatory (Table 1). The signaling members of the IL-1 family share a highly conserved gene sequence and structure and (except for IL-18 and IL-33) are clustered on human chromosome 2 (Sims and Smith, 2010). Due to these similarities, the genomic identification of IL-1 family members largely preceded the discovery of their function. Aside from IL-1 receptor antagonist (IL-1Ra), all other IL-1 family cytokines lack a secretion signal peptide and either require cleaving and activation or are active in their precursor form. Signaling typically occurs when cytokines bind their primary receptor and recruit a co-receptor, which induces signaling through the Toll/interleukin 1 receptor (TIR) domain resulting in mitogen-activated protein kinase (MAPK) and nuclear factor kappa B (NF-κB) activation (Fields et al., 2019). In this review, we describe each IL-1 subfamily (IL-1, IL-18, and IL-36) and investigate the mechanism of induction and functional role of each subfamily member within the context of fungal disease.

**TABLE 1:** Nomenclature and function of IL-1 family members.

Cytokine		Primary receptor		Co-receptor		Effect
Name	Alternate name(s)	Name	Alternate name(s)	Name	Alternate name(s)	
IL-1α	IL-1F1	IL-1R1/IL-1R2 (Decoy)		IL-1RAcP	IL-1R3	Pro-inflammatory
IL-1β	IL-1F2	IL-1R1/IL-1R2 (Decoy)		IL-1RAcP	IL-1R3	Pro-inflammatory
IL-1Ra	IL-1F3	IL-1R1				Anti-inflammatory
IL-33	IL-1F11	IL-1R4	ST2	IL-1RAcP	IL-1R3	Pro-inflammatory
IL-18	IL-1F4	IL-18Rα	IL-1R5	IL-18Rβ	IL-1R7	Pro-inflammatory
IL-37	IL-1F7	IL-18Rα	IL-1R5	IL-1R8	SIGIRR	Anti-inflammatory
IL-36α	IL-1F6	IL-36R	IL-1Rrp2/IL-1R6	IL-1RAcP	IL-1R3	Pro-inflammatory
IL-36β	IL-1F7	IL-36R	IL-1Rrp2/IL-1R6	IL-1RAcP	IL-1R3	Pro-inflammatory
IL-36γ	IL-1F8	IL-36R	IL-1Rrp2/IL-1R6	IL-1RAcP	IL-1R3	Pro-inflammatory
IL-36Ra	IL-1F5	IL-36R	IL-1Rrp2/IL-1R6			Anti-inflammatory
IL-38	IL-1F10	IL-36R	IL-1Rrp2/IL-1R6			Anti-inflammatory

## The IL-1 Subfamily

Interleukin-1 was the first interleukin to be identified and represented a huge leap forward in immunology. The IL-1 gene cluster encodes the pro-inflammatory cytokines IL-1α and IL-1β, and anti-inflammatory receptor antagonist IL-1Ra (Garlanda et al., 2013). IL-1α/β bind their receptor IL-1R1, which recruits IL-1RAcP and permits signaling through both receptor’s TIR domain (Fields et al., 2019). This domain is well conserved throughout IL-1 and Toll-like receptor (TLR) signaling where it drives inflammation (Heguy et al., 1992). IL-1α/β are recognized as major inflammatory cytokines that mediate innate and adaptive immunity, and also general health. IL-33, a recent addition to the IL-1 subfamily, was discovered in 2005 and signals through its receptor IL-1R4 (formerly ST2) in complex with IL-1RAcP. IL-33 has many functional capabilities which influence barrier integrity and inflammation (Mehraj et al., 2016). IL-33 signaling has been implicated in numerous allergic-type diseases and, along with IL-1α/β, has recently been shown as a mediator of adaptive immunity (Kamijo et al., 2013).

### IL-1 Subfamily Expression and Processing

Although IL-1α and IL-1β signal through the same receptor and have similar biological activities, these two pro-inflammatory cytokines differ in several aspects. IL-1α is constitutively expressed in epithelial and mesenchymal cell types and expression increases in response to growth factors, inflammation or stress-associated stimuli (Di Paolo and Shayakhmetov, 2016). Here, IL-1α is released from cells during damage or necrosis (Chen et al., 2007). IL-1α also possesses a nuclear localization signal and can interact with histone acetyltransferase complexes to mediate transcription of cytokines including IL-6 and IL-8 (Werman et al., 2004). However, nuclear translocation of IL-1α during apoptosis inactivates IL-1α signaling, likely sequestering it and blocking inflammatory effects (Cohen et al., 2010). IL-1α also functions as an active membrane bound precursor promoting inflammation through IL-1R1 binding. Membrane-associated IL-1α is present on the surface of numerous immune cells including macrophages (Kurt-Jones et al., 1985), monocytes, and B lymphocytes (Zola et al., 1993).

Interleukin-1β is mainly produced by mononuclear phagocytes as an inactive precursor and activated via a two-step process. During the initial priming step, pro-IL-1β is induced by the recognition of pathogen-associated molecular patterns (PAMPs) by pattern recognition receptors (PRR) (Takeuchi and Akira, 2010). Activation requires pro-IL-1β cleavage by the intracellular cysteine protease caspase-1 and is regulated by inflammasomes (Franchi et al., 2009). Although caspase-1 is the main protease responsible for pro-IL-1β activation, Fas stimulation is also able to induce secretion of biologically active IL-1β from caspase-1-deficient murine macrophages (Bossaller et al., 2012). Once activated, IL-1β is secreted through one or more non-conventional secretory pathways (Lopez-Castejon and Brough, 2011). Here, another role for caspase-1 has been described in that it can cleave the pore forming toxin gasdermin D, which induces pore formation in the plasma membrane, and pyroptosis to enhance IL-1β release (Heilig et al., 2018). Importantly, the release of pro-IL-1β upon membrane disruption has functional consequences. Multiple proteases, mainly derived from neutrophils and mast cells, can cleave and activate pro-IL-1β in the extracellular environment which drives inflammation (Stehlik, 2009).

Interleukin-33 is constitutively expressed in multiple cell types but is mainly found in fibroblasts, epithelial and endothelial cells (Moussion et al., 2008), with expression further increasing during inflammation (Liew et al., 2016). In macrophages, IL-33 induction was dependent on glutaredoxin-1/TRAF6 and NF-κB signaling (Weinberg et al., 2019). Similar to IL-1α, IL-33 has dual functions acting as a transcriptional repressor of NF-κB following nuclear localization (Ali et al., 2011) and enhancing inflammation after being released from damaged or necrotizing cells (Moussion et al., 2008). Again like IL-1α, IL-33 is also sequestered during apoptosis (Bessa et al., 2014). Interestingly, caspase-1 IL-33 cleavage attenuated inflammation (Luthi et al., 2009), while neutrophil proteases enhanced biological activity (Lefrancais et al., 2012). Following the release of IL-1α and IL-33, and the release and activation of IL-1β, the cytokines drive potent immunological functions.

### The Immunological Function of the IL-1 Subfamily

The first discovered IL-1 cytokine was named hemopoietin-1 after its signaling drove myeloid “emergency” responses (Pietras et al., 2016). Since then, the sub-family has expanded and IL-1α/β are now known to promote myelopoiesis and inflammation, lead to the release of antimicrobial compounds, and mediate immunity (Dinarello, 2018). Following the release of IL-1α and active IL-1β, the cytokines bind their receptor IL-1R1 and drive MAPK and NF-κB signaling through the myeloid differentiation primary response (MyD88) adaptor protein (Cohen, 2014). Two mechanisms antagonize the effects of IL-1α/β. Firstly, IL-1Ra binds IL-1R1 to prevent IL-1α/β-IL-1R1 interactions. The importance of IL-1Ra is highlighted in IL-1Ra deficient mice, which spontaneously develop Th17-associated rheumatoid arthritis (Koenders et al., 2008). Secondly, IL-1R2 acts as a decoy receptor and lacks a TIR domain, therefore blocking downstream signaling. Overexpressing IL-1R2 in mice reduced inflammation in numerous IL-1-induced inflammatory diseases (Peters et al., 2013).

While IL-1α/β have central roles in driving inflammation and mediating immune responses, each cytokine possesses distinct function. IL-1α acts as an alarmin and is the principal trigger of inflammation following cell membrane disruption or cellular necrosis. In this context, constitutively expressed IL-1α is released from cells and drives neutrophil recruitment and local inflammation (Chen et al., 2007; Eigenbrod et al., 2008). IL-1β signaling promotes the recruitment of monocytes, macrophages, and neutrophils; enhanced phagocytosis and killing; increased reactive oxygen species/nitrogen oxide synthase (ROS/NOS) production; and Th1 and Th17 immunity (Garlanda et al., 2013; Altmeier et al., 2016; Dinarello, 2018). Mice deficient in caspase-1 were protected from Th1 and Th17-associated experimental autoimmune encephalomyelitis (Gris et al., 2010) and mice deficient in IL-1Ra developed spontaneous Th17-associated autoimmunity (Horai et al., 2000). IL-1 is also associated with numerous autoinflammatory diseases, with blockade of IL-1 signaling rapidly improving symptoms (Dinarello et al., 2012). Interestingly, even when the autoimmune disease is primarily tumor necrosis factor (TNF)/IL-6 driven (as in rheumatoid arthritis), blocking IL-1β signaling reduced disease severity (Dinarello, 2011). This highlights the central role of IL-1α/β mediating immunity and health.

Interleukin-33 acts in a similar fashion to IL-1α, being released from barrier cells and functioning as a damage-associated molecular pattern (DAMP). Here, IL-33 activates T-cells directly through PRR/IL-1R4 binding or indirectly through local inflammatory responses. Immune cells such as mast cells, basophils, dendritic cells, macrophages, natural killer cells, and Th2 cells are receptive to IL-33 and express IL-1R4 (Martin and Martin, 2016). The soluble form of IL-1R4 sequesters “off-target” IL-33 and antagonizes IL-33 activity (Kakkar and Lee, 2008). IL-33 was originally identified as driving allergic and anti-helminthic immunity but is also a key mediator of adaptive immunity. During acute infection, IL-33 promotes tissue remodeling and drives Th1, Th2, and T regulatory (Treg) responses. In contrast, during chronic infection, IL-33 enhanced local inflammation, tissue damage and fibrosis (Li et al., 2014). IL-33 signaling has been implicated in numerous diseases associated with exacerbated inflammation including allergic-asthma (Salter et al., 2016), Crohn’s disease (Pastorelli et al., 2010), and rheumatoid arthritis (Tang et al., 2013). While the broad and potent consequences of IL-1α/β and IL-33 signaling are known, the induction of these cytokines during fungal infection is less clear.

### Fungal Induction of the IL-1 Subfamily

Numerous fungal pathogens infect mucosal barrier sites and systemic disease often results from a loss in barrier integrity. It is therefore unsurprising that IL-1α/β and IL-33 have been implicated in anti-fungal immunity. All three cytokines are induced following *Aspergillus fumigatus* infection, a pathogen that typically infects mucosal surfaces. IL-1α expression in the lung is positively correlated with *A. fumigatus* strain virulence (Caffrey-Carr et al., 2017). This is likely a result of conidial germination and subsequent damage to mucosal barriers. IL-1β was rapidly induced and sustained for 2 days in an *A. fumigatus* keratitis model. Here, IL-1β induction was dependent on Dectin-1 signaling and c-Jun N-terminal kinase (JNK) phosphorylation (Yuan et al., 2017). Numerous PRRs that induce IL-1β signaling, including C-type lectin-like receptors (CLRs) and TLRs, are involved during *A. fumigatus* infection (Steele et al., 2005). The requirement of CLRs for IL-33 induction appears more complex. During acute *A. fumigatus* infection the induction of IL-33 occurred independently of Dectin-1 (Garth et al., 2017), while induction during chronic allergic-type infection required Dectin-1 (Lilly et al., 2012).

*Candida albicans* infection also results in IL-1α/β and IL-33 induction. IL-1α induction has been described in oral and vaginal epithelial cells (Steele and Fidel, 2002) and required membrane disruption and Ca^2+^ influx (Gross et al., 2012). Here, the peptide toxin candidalysin, secreted when *C. albicans* forms hyphae, disrupts host membranes resulting in IL-1α release (Moyes et al., 2016). IL-33 is also induced during *C. albicans* infection at barrier sites (Le et al., 2012) and likely requires candidalysin expression in a similar manner to IL-1α. The induction of IL-1β in monocytes was only achieved with live *Candida* and does not require CLR involvement (Castro et al., 1996). In contrast, dendritic cells (that require two signals for IL-1β induction and activation) were dependent on spleen tyrosine kinase (Syk) signaling for IL-1β induction during *C. albicans* infection (Gross et al., 2009). In macrophages, IL-1β is induced by *C. albicans, C. tropicalis*, and *C. krusei*, although the requirement for CLRs is disputed (Joly et al., 2009; Kasper et al., 2018). As such, there are several mechanisms by which *Candida* induce inflammasome activation and IL-1β release (Camilli et al., 2020). One particularly interesting mechanism involves the non-tyrosine kinase Tec which was found to activate non-canonical caspase-8 exclusively following fungal challenge (Zwolanek et al., 2014). IL-1α/β induction during mucosal *C. albicans* infection occurs through NF-κB and a biphasic MAPK response. Activation of NF-κB and the first MAPK c-Jun phase was dependent on fungal PAMP recognition, while the second MAPK MKP1/c-Fos phase was dependent on hyphae formation and fungal burden. This identifies an interesting mechanism by which the host may detect the switch from commensalism to pathogenicity (Moyes et al., 2010). A similar biphasic recognition mechanism has not yet been described for other fungal infections, although such a mechanism may also differentiate between *A. fumigatus* conidia and hyphae during infection.

Interleukin-1 subfamily members are also induced by *Cryptococcus neoformans*, *Paracoccidioides brasiliensis*, and *Paracoccidioides lutzii* [the causative agents of paracoccidioidomycosis (PCM)], and *Sporothrix schenckii* (the causative agent of sporotrichosis). IL-1α/β and IL-33 are induced in both mucosal and systemic compartments during *C. neoformans* infection (Flaczyk et al., 2013; Alvarez et al., 2019). During *P. brasiliensis* challenge, IL-1β was induced in human monocytes, macrophages and plasmacytoid dendritic cells (pDC). As was found with *Candida*, CLRs and Syk signaling were required for pDC IL-1β induction (Kurokawa et al., 2007; Preite et al., 2018). IL-1β release from macrophages was caspase-1 dependent; however, a non-canonical IL-1β processing pathway requiring Dectin-1 and caspase-8 activity has been described (Ketelut-Carneiro et al., 2018). *P. brasiliensis* challenge also induced IL-1α release through a macrophage-derived IFN-β/procaspase-11 pathway which enhanced pore-mediated cell lysis (Ketelut-Carneiro et al., 2019). Patients with severe PCM possessed high levels of IL-1β, IL-33, and IL-1R4 in serum which decreased following anti-fungal therapy (Silva et al., 1995; Alves et al., 2018). Recently, IL-1β has been implicated in *S. schenckii* infection. Here, IL-1β was induced in a caspase-1 dependent manner with increased IL-1β correlating with higher fungal burden (Goncalves et al., 2015). While it is clear the IL-1 subfamily is induced by fungal pathogens and contributes to fungal immunity, the sequence and mechanism of IL-1 subfamily induction during fungal challenge is still unclear.

### The Role of the IL-1 Subfamily in Fungal Immunology

Interleukin-1α/β drive crucial immune mechanisms and are central mediators of immunity. Following their induction during fungal infection, both IL-1α/β play a key role orchestrating the immune response. While IL-33 is induced during fungal disease, its functional role is not yet fully delineated. Much of our understanding of the IL-1 subfamily during fungal disease has been revealed using mice deficient in IL-1 components. Mice lacking IL-1R1 exhibit reduced neutrophil recruitment and emergency granulopoieses following oropharyngeal candidiasis (OPC) challenge. Here, both IL-1β from hematopoietic cells and IL-1α from non-hematopoietic cells promoted neutrophil recruitment (Altmeier et al., 2016). However, neutrophil recruitment is not always protective, since in vulvovaginal candidiasis (VVC) IL-1α promotes damaging inflammation and neutrophil recruitment (Barousse et al., 2004). In agreement, mutations increasing Nod-like receptor family pyrin domain containing 3 (NLRP3) inflammasome activity are associated with VVC in patients (Jaeger et al., 2016; Roselletti et al., 2017).

Mice deficient in IL-1α or IL-1β were more susceptible to systemic *C. albicans* infection, with mice lacking both IL-1α/β possessing the highest fungal burdens and lowest survival. Interestingly, IL-1α/β deficient mice continued to possess higher fungal burdens in a neutropenic model of systemic *C. albicans* infection, suggesting the role of IL-1α/β is not just in neutrophil recruitment. Instead, IL-1α was found to activate macrophages and IL-1β enhanced neutrophil killing, and both IL-1α and IL-1β were involved in the development of IFN-γ Th1 and IL-10 Th2 responses (Vonk et al., 2006). A role for epithelial and hematopoietic IL-1α/β has also been described to promote essential Th17 immunity during mucosal *Candida* infection (Bishu et al., 2014; Verma et al., 2017, 2018). In patients, mutations in caspase recruitment domain-containing protein 9 (CARD9) or TLR1 (pathways that lead to IL-1β induction) resulted in defective Th17 responses and increased *Candida* susceptibility (Glocker et al., 2009; Plantinga et al., 2012). Th17 immunity is also vital during *Coccidioides* infection. Here, Th17 responses were dependent on CARD9 and IL-1R1, with IL-1R1 deficient mice lacking Th17 immunity and being highly susceptible to *C. immitis* infection (Hung et al., 2014; Viriyakosol et al., 2018). IL-1R1 deficient mice are highly susceptible to *C. neoformans* infection; however, susceptibility was associated with the induction of harmful Th2 immunity (Shourian et al., 2017).

Both IL-1α and IL-1β drive protective immunity during PCM. *In vivo* models of *P. brasiliensis* infection determined mice with impaired IL-1β responses had higher fungal burden and a dysregulated inflammatory response. IL-1α promoted local inflammatory responses, nitric oxide production and Th17 immunity (Ketelut-Carneiro et al., 2018, 2019). Whilst signaling through IL-1R1 is required for protective immunity against pulmonary *A. fumigatus* infection; unlike *Candida* and *P. brasiliensis* infection, only IL-1α was required. Here, IL-1α promoted neutrophil and macrophage recruitment through monocyte-induced CXCL1 signaling. Administration of CXCL1 partially restored neutrophil recruitment in IL-1R1 deficient mice (Caffrey et al., 2015), while mice lacking inflammasome components, and thus IL-1β activation, cleared *A. fumigatus* infection (Caffrey et al., 2015). However, IL-1β may play a key role during systemically disseminated *Aspergillus* infection, as was described with systemic *Candida* infection (Vonk et al., 2006). The Dectin-1 Y238X polymorphism, which results in diminished Dectin-1 activity and reduced IL-1β responses, significantly enhanced patient’s *Aspergillus* and *Candida* susceptibility (Plantinga et al., 2009; Cunha et al., 2010).

While the potent effects of IL-1α/β are clear during fungal disease, the effect of IL-1Ra and IL-1R2 are less well explored. Patients with severe fungal-sensitized asthma have higher levels of IL-1α/β and IL-1Ra, indicating the antagonist is involved. In an animal model of fungal-sensitized asthma, a lack of IL-1Ra enhanced Th1 and Th17 immunity, increased neutrophil recruitment and exacerbated disease. Treatment with recombinant IL-1Ra (Anakinra) reduced these responses and resolved disease. In the same model, IL-1R1 deficient mice displayed improved lung function, suggesting IL-1α/β enhance chronic disease (Godwin et al., 2019). IL-1Ra also has a protective role in VVC by restraining activation of damage-enhancing NLRP3 through an IL-22/NLR family CARD domain-containing protein 4 (NLRC4)/IL-1Ra pathway. Here, mice deficient in IL-1Ra displayed enhanced disease that could be rescued with the administration of Anakinra (Borghi et al., 2015). While the role of IL-1Ra and IL-1R2 in balancing IL-1 agonist activity during fungal disease requires further investigation, early indications suggest IL-1Ra and IL-1R2 play important roles in mediating and resolving potentially damaging inflammation during acute and chronic disease.

The role of IL-33 signaling is now being explored in the context of fungal infection. During *C. neoformans* infection, IL-1α/β drives protective Th17 immunity and reduces Treg responses, while IL-33 promotes the suppressive function of Treg cells (Alvarez et al., 2019) and enhances IL-5 and IL-13-derived Th2 immunity. This IL-33-Th2 response resulted in increased fungal burdens and reduced survival in murine models (Flaczyk et al., 2013). A deleterious role for IL-33 has also been described during acute and chronic *A. fumigatus* disease. Mice deficient in IL-33 produced more IL-17A and IL-22 and displayed enhanced fungal clearance when challenged with acute *A. fumigatus* infection. Furthermore, blocking IL-1R4 in a model of *Aspergillus*-sensitized asthma improved airway hyperresponsiveness and fibrosis (Ramaprakash et al., 2011). In contrast, pre-treatment of mice with IL-33 prior to peritoneal and systemic *C. albicans* infection resulted in fungal clearance and improved survival. Here, IL-33 enhanced the recruitment of neutrophils and increased their killing capability while also mediating T-cell tolerance (Le et al., 2012; Tran et al., 2015).

### Therapeutic Potential of the IL-1 Subfamily

The activity of IL-1α, IL-1β, and IL-33 must be carefully regulated. Protection from pathogen-derived and autoinflammatory diseases likely involves a balance of IL-1 subfamily agonist and antagonist activity. Excessive IL-1/Th17 signaling results in numerous diseases including asthma, chronic obstructive pulmonary disease (Gurczynski and Moore, 2018), inflammatory bowel disease (IBD), and psoriasis (Beringer et al., 2016). As such, the IL-1/Th17 pathway has been therapeutically targeted. Anakinra blocks IL-1α/β activity and is used as a therapy in multiple inflammatory diseases (Dinarello et al., 2012). Similarly, targeting IL-1 signaling components is known to reduce inflammation and pathology in fungal-induced respiratory disease (Gresnigt et al., 2016; Griffiths et al., 2018; Godwin et al., 2019). Alongside Anakinra, numerous other IL-1 therapeutic strategies have been investigated. Examples include EBI-005, an IL-1R1 antagonist (Hou et al., 2013), Rilonacept (trade name Arcalyst), a decoy IL-1R1 (Economides et al., 2003), SL1067, a DNA aptamer that disrupts IL-1α (Ren et al., 2017), AF10847, a peptide inhibitor of IL-1R1 (Vigers et al., 2000), and AMG108, an antibody inhibitor of IL-1R1 (Cohen et al., 2011). IL-33 also has therapeutic potential and was effective in supporting immune defenses prior to *C. albicans* infection (Tran et al., 2015). The exogenous administration of IL-1 sub-family agonists or antagonists has potential to enhance protective anti-fungal immunity or resolve excessive, damaging immune responses. Another therapeutic route may involve the modulation of receptors or proteins that regulate IL-1 sub-family signaling. A clear link between CLRs and IL-1α/β signaling exists, as evidenced by CARD9-deficient patient’s reduced IL-1α/β expression and susceptibility to fungal infection (Drummond et al., 2015). Furthermore, Dectin-1-induced IL-1β protected against mucosal *Candida* infections becoming systemic and lethal (Hise et al., 2009). In summary, modulating mediators of IL-1 sub-family induction, translation and processing may provide novel therapeutic targets in the fight against fungal infections.

## The IL-18 Subfamily

Interleukin-18 was first described in 1989 as IFN-γ inducing factor before it was re-named when its function was described as pro-inflammatory (Dinarello, 2018). IL-18 and IL-1β share similar structures and signaling pathways, and are produced as inactive precursors requiring caspase-1 activation; however, the two cytokines are functionally distinct (Dinarello, 2019). IL-18 forms a low affinity signaling complex by binding IL-18Rα, the ligand for mature IL-18. A high affinity signaling complex is formed in cells that also express the co-receptor IL-18Rβ (Kaplanski, 2018). Similar to IL-1α/β, signal transduction requires the TIR domain and drives NF-κB and MAPK signaling (Hoshino et al., 1999; Wyman et al., 2002). IL-18 is regulated by IL-18 binding protein (IL-18BP), a soluble protein with high affinity for IL-18 (Krumm et al., 2014), and IL-37 which binds IL-18Rα and inhibits recruitment of IL-18Rβ. Moreover, IL-37 binding of IL-18Rα induces recruitment of IL-1R8 (also called SIGIRR), which induces anti-inflammatory signaling (Nakanishi, 2018).

### IL-18 Subfamily Expression and Processing

Interleukin-18 is a pleiotropic cytokine that plays a central role in immunity (Novick et al., 2013). Like IL-1β, IL-18 is produced as an inactive precursor and must be processed by the inflammasome/caspase-1 complex in order to be activated and secreted (Franchi et al., 2009). However, unlike IL-1β, IL-18 is constitutively expressed by several hemopoietic and non-hemopoietic cells, including macrophages, dendritic cells, Kupffer cells, keratinocytes, osteoblasts, adrenal cortex cells, intestinal epithelial cells, microglial cells, and synovial fibroblasts (Gracie et al., 2003). Fas stimulation was able to induce secretion of biologically active IL-18 in caspase-1-deficient murine macrophages (Bossaller et al., 2012). In addition, proteases secreted by the inflammatory cell infiltrate contribute to inflammation via activation of IL-18 (Omoto et al., 2006, 2010). IL-18 can be secreted from monocytes and macrophages in its active form (Fantuzzi et al., 1999), or released from dying endothelial and epithelial cells in its precursor form before being activated outside the cell (Sugawara et al., 2001). IL-37 acts as an IL-18 antagonist and is found in many human tissues (Dinarello et al., 2016), although more specific roles for IL-37 reducing inflammation in the lung, spleen, plasma and in dendritic cells have been described (Nold et al., 2010). IL-37 is similar to IL-1α and IL-33, functioning as a dual-action cytokine that does not require activation (Li et al., 2015), however, caspase-1 processing was required for IL-37 to translocate to the nucleus to modulate transcription. IL-37 can be released in a processed or precursor form, resulting from exogenous administration of ATP (Bulau et al., 2014). IL-18BP also acts as an IL-18 antagonist and contains a signal peptide that directs secretion into the extracellular environment (Novick et al., 2001).

### The Immunological Function of the IL-18 Subfamily

Following induction and activation, IL-18 signaling drives inflammation and promotes innate and adaptive immunity. Accordingly, IL-18 signaling can direct robust anti-pathogen immunity but is also associated with numerous autoinflammatory diseases. A clear role for IL-18 promoting Th1 immunity through IFN-γ induction has been described during models of viral and bacterial infection. In addition, IL-18BP is targeted by viruses to reduce host-protective IL-18 mediated immunity (Born et al., 2000; Reading and Smith, 2003). Mice deficient in IL-18 components were highly susceptible to viral and bacterial challenge (Mastroeni et al., 1999; Kinjo et al., 2002; Freeman et al., 2015). However, IL-18 can only induce IFN-γ and Th1 immunity in combination with IL-12 or IL-15 signaling; IL-18 signaling alone enhances Th2 immunity (Nakanishi et al., 2001), which is typically detrimental for fungal clearance. An IL-18 synergy primarily with IL-12 but also IL-15 exists with each cytokine modulating the others transcription, expression, and receptor expression (Fantuzzi et al., 1999). Recently, IL-18 has also been shown to drive IL-17 production from γδ T-cells and promote Th17 responses (Sutton et al., 2012), modulate adhesion molecules in endothelium (Carrascal et al., 2003), promote nitric oxide (NO) synthesis (critical for viral and bacterial killing) and enhance production of numerous chemokines (Kaplanski, 2018). IL-18 contributes to autoimmune disease including Type-1 diabetes, psoriasis, IBD, asthma and numerous myocardial and kidney diseases (Nakanishi et al., 2001; Garlanda et al., 2013). Here, increased expression of IL-18 and IL-18BP has been observed suggesting that poor agonist/antagonist balance may result in disease. In agreement, the majority of IL-18 associated autoimmune diseases result from excess Th2 immune responses (Monteleone et al., 1999; Tanaka et al., 2001; Gerdes et al., 2002), potentially from IL-18 activity without IL-12 or IL-15.

Interleukin-37 has a broad anti-inflammatory effect limiting IL-18 responses. Reducing IL-37 in human cells increased the production of cytokines (IL-1β/TNF/IL-6) induced by IL-1 and TLRs (Nold et al., 2010). IL-37 is the only member of the IL-1 family not to have a mouse homolog, as such *in vivo* work is only possible with transgenic IL-37 models. Here, the anti-inflammatory effects of IL-37 have been demonstrated to reduce colitis, metabolic syndrome, acute lung injury, myocardial infarction, and asthma (Wu et al., 2014; Li et al., 2015). IL-18BP also acts to reduce IL-18 agonist activity and an imbalance of IL-18 and IL-18BP has been described in Wegener’s granulomatosis and systemic lupus erythematosus (Novick et al., 2009, 2010). Administration of IL-18BP reduced inflammation in a model of rheumatoid arthritis; however, at high concentrations IL-18BP also bound IL-37 and the anti-inflammatory effect was lost (Banda et al., 2003). The functional implications of IL-18 signaling driving inflammation, innate and adaptive immune responses in autoimmune and anti-pathogen disease are clear. However, the induction, function, and balance of the IL-18 subfamily during fungal disease is less well explored.

### Fungal Induction of the IL-18 Subfamily

Interleukin-18 is a crucial cytokine that mediates innate and adaptive immunity and likely plays a key role during fungal infection. The cytokine is expressed in cells of mesenchymal origin and hematopoietic cells and therefore may share functions with both IL-1α and IL-1β. In agreement, the induction of IL-18 has been observed during systemic and mucosal fungal infections. IL-18 was induced following acute *Aspergillus* challenge and during a chronic model of fungal-sensitized asthma (Cenci et al., 1997, 1998). A recent study revealed IL-18 expression increased rapidly following *Aspergillus* challenge peaking at 24 h before resolving over the next 48 h (Cheng et al., 2020). However, the signaling events leading to IL-18 induction in these models has not been defined. *Aspergillus* conidia frequently interact with barrier surfaces and systemic disease arises from germination in this setting. Understanding the induction of IL-18 as *Aspergillus* conidia persist, germinate, and promote disease may lead to important findings.

Interleukin-18 is also induced during *Candida* infection. Oral epithelial cells constitutively expressed IL-18 mRNA and precursor IL-18. During *C. albicans* challenge expression of IL-18 mRNA and pre-IL-18 was reduced while active IL-18 was released in a caspase-1 dependent manner (Rouabhia et al., 2002). A similar effect was seen in a model of human oral mucosa where *C. albicans* challenge resulted in active IL-18 expression. In agreement, patients with oral candidiasis possess increased levels of active IL-18 in saliva samples (Tardif et al., 2004). There is limited evidence describing the signaling events required for IL-18 induction in fungal disease. Recent investigation determined that Dectin-1 signaling and activation of the non-canonical NF-κB subunit RelB resulted in IL-18 induction (Shen et al., 2020); however, little else is known. Investigating the mechanism of IL-18 induction during mucosal and systemic *Candida* disease may provide insight into protective anti-*Candida* immunity. Aside from the induction of IL-18 during *Aspergillus* and *Candida* disease, IL-18 has been functionally implicated in *P. brasiliensis* infection (Panagio et al., 2008; Alves et al., 2018), *S. schenckii* infection (Goncalves et al., 2015), and *C. neoformans* infection (Kawakami et al., 2000b; Wang et al., 2011), suggesting at the very least a general role in host immune response. Whether IL-37 and IL-18BP are induced during fungal disease currently unclear; however, increased serum IL-37 was identified in PCM patients with severe disease (Alves et al., 2018).

### The Role of the IL-18 Subfamily in Fungal Immunology

While the induction of IL-18 requires further investigation, the functional consequences of IL-18 signaling during fungal disease highlight the importance of this IL-1 subfamily member. During acute *Aspergillus* lung infection, IL-18 promoted protective immunity and enhanced Th1 immunity and neutrophil recruitment in concert with IL-12 and IFN-γ (Blease et al., 2001). Skewing toward Th2 immunity during acute *Aspergillus* infection is non-protective and promotes chronic disease (Cenci et al., 1998). A study of acute *Aspergillus* infection determined 72 h after infection IL-18 mediates protection independently of IFN-γ, suggesting that the IL-18/IFN-γ axis occurs rapidly and IL-18 continues to mediate immunity independently of IFN-γ if the infection persists (Brieland et al., 2001). However, in immunocompromised models, IFN-γ was essential throughout infection and exogenous administration of IFN-γ was consistently protective (Nagai et al., 1995; Cenci et al., 1997, 1998). In an *Aspergillus*-sensitized asthma model, IL-18 was again found to be protective and acted without IL-12 or IFN-γ to enhance *Aspergillus* clearance. Depleting IL-18 in this model increased fungal burden and resulted in persistent airway hyperactivity and fibrosis (Blease et al., 2001). These results suggest IL-18 is protective during both acute and chronic *Aspergillus* disease but provides protection through distinct mechanisms.

Interleukin-18 is also vital for protection during *Candida* infection. Similar to *Aspergillus* infection, IL-18 induced protective Th1 immunity against *Candida*. Mice deficient in caspase-1 displayed reduced Th1 responses and were susceptible to *Candida* challenge. Here, the exogenous administration of IL-18, without IL-1β, restored Th1 responses and protection (Mencacci et al., 2000). In addition, the exogenous administration of IL-18BP reduced IFN-γ-derived Th1 immunity in human whole blood cultures (Netea et al., 2002) and in mice (Fantuzzi et al., 1999). In agreement, administration of anti-IL-18 antibodies prior to systemic *Candida* infection depleted IFN-γ responses and enhanced fungal disease (Stuyt et al., 2002), whereas increasing IL-18 enhanced IFN-γ and Th1 responses which ultimately promoted protection (Stuyt et al., 2004). Interestingly, the role for IL-18 mediating neutrophil recruitment is unclear with one study suggesting IL-18 is uncoupled from neutrophil recruitment (Netea et al., 2003). This aligns with the protective role of IL-18 during VVC, where neutrophil recruitment enhances disease. VVC disease results from unrestrained NLRP3 activation/continuous IL-1β stimulation and is regulated by the IL-22/NLR4C axis. In this setting, IL-18 acts in a cross-circuit with IL-22 with both cytokines regulating each other and reducing NLRP3 activity (Borghi et al., 2019). IL-18 appears to be broadly protective during *Candida* infection, although, as was found with *Aspergillus*, IL-18 acts through many distinct mechanisms to promote immunity.

Interleukin-18 signaling has also been described during *C. neoformans* and *P. brasiliensis* infection. IL-18 deficient mice exhibit increased *C. neoformans* fungal burdens and reduced IFN-γ/IL-12 responses (Kawakami et al., 2000a, b). In addition, IL-18R deficient mice were more susceptible to *C neoforman*s than IL-1R deficient mice, suggesting IL-18 and not IL-1α/β signaling mediates *C. neoformans* immunity (Wang et al., 2011). While IFN-γ and Th1 immunity are vital during PCM, the role of IL-18 is controversial. IL-18 deficient mice on a BALB/c background were protected from PCM challenge and displayed increased survival and reduced fungal burden (Panagio et al., 2008). In contrast, IL-18 deficient mice on a C57BL/6 background were susceptible to PCM and displayed enhanced fungal burdens (Ketelut-Carneiro et al., 2015). In patients with PCM, increased IL-18 in serum correlated with more severe forms of disease (Corvino et al., 2007), suggesting the C57BL/6 mouse model may be more appropriate.

A role for IL-37 has been described in a murine model of pulmonary aspergillosis. Here, administration of IL-37 decreased NLRP3 activity and IL-1β expression through the SIGIRR signaling pathway and resulted in reduced inflammatory cell recruitment. This reduced tissue damage during acute *Aspergillus* infection, and dampened adaptive responses in chronic *Aspergillus* infections (Moretti et al., 2014). SIGIRR signaling has been described to prevent lethal dysregulated IL-1 dependent Th17 responses in fungal disease (Warris et al., 2005). The induction and function of IL-18BP and IL-37 during fungal disease requires investigation but these antagonists may provide important functions mediating IL-18 signaling, enhancing immune responses, and resolving inflammatory effect. Numerous viruses target IL-18BP as an immune evasion strategy; whether fungi can do the same would be interesting to determine.

### Therapeutic Potential of the IL-18 Subfamily

The exogenous administration of IL-18 enhances immunity in systemic models of *C. albicans* infection. This strategy may also provide protection during *Aspergillus* and *Cryptococcus* infection where IL-18 responses also confer protection. IL-18 therapy may be targeted at barrier sites or systemically once any difference in IL-18 induction and function at these two sites is determined. Although the exogenous administration of IL-18BP and anti-IL-18 antibodies enhanced acute fungal susceptibility, both these IL-18 depleting strategies promote the resolution of autoinflammatory disease and improve chronic autoimmune disease (Kanai et al., 2001; Sivakumar et al., 2002). Although no IL-18 therapeutics are currently licensed, IL-18BP therapy was examined in rheumatoid arthritis and psoriasis patients with positive tolerance and safety profiles (Tak et al., 2006). IL-37 has clear therapeutic potential as the administration of IL-37 during *Aspergillus* infection promoted beneficial inflammatory resolution in both acute and chronic disease (Moretti et al., 2014).

## The IL-36 Subfamily

Interleukin-36 is a recent addition to the IL-1 superfamily that was discovered and characterized 20 years ago. Initially, IL-36 was thought to be similar to IL-1 as the two members of the IL-1 family shared similar gene sequences, exon-intron arrangements and predicted protein structure (Smith et al., 2000). Intriguingly, however, these new IL-36 cytokines were unable to bind IL-1R or any known orphan receptors in the IL-1 superfamily (O’Neill and Dinarello, 2000). Shortly after, two studies determined that IL-36 cytokines signal through a complex of IL-36R and IL-1RAcP leading to NF-κB and MAPK activation, and IL-6 and IL-8 production (Debets et al., 2001; Towne et al., 2004). We now know the IL-36 subfamily is comprised of four IL-36 isoforms, three agonists IL-36α, IL-36β, and IL-36γ driving proinflammatory functions (Towne et al., 2004), and the IL-36Ra antagonist mediating inflammation (Debets et al., 2001). It is worth noting that although the IL-36 subfamily was renamed in 2010, the previous nomenclature is still frequently encountered. IL-36α, IL-36β, IL-36γ, and IL-36Ra were known as IL-1F6, IL-1F8, IL-1F9, and IL-1F5, respectively, and IL-36R was named IL-1Rrp2 (Dinarello et al., 2010). IL-38 also belongs to the IL-36 sub-family, signals through IL-36R and functions as a receptor antagonist similar to IL-36Ra (van de Veerdonk et al., 2012).

### IL-36 Subfamily Expression and Processing

The IL-36 subfamily plays a key role driving immune responses at mucosal barriers in the skin, respiratory tract and intestine and are the only cytokines constitutively expressed in epithelium (D’Erme et al., 2015). This location-specific expression is not found with IL-1 or IL-18 (Gresnigt and van de Veerdonk, 2013). IL-36 agonist expression is predominantly restricted to epithelial cells (Towne et al., 2004; Blumberg et al., 2007), although expression has also been observed in macrophages, dendritic cells and monocytes (Smith et al., 2000; Vigne et al., 2011; Mutamba et al., 2012; Boutet et al., 2016). IL-36R transcripts are highly prevalent in keratinocytes and epithelial cell types (Kumar et al., 2000; Towne et al., 2004), and have been found in naïve CD4 + T-cell subsets (Vigne et al., 2012), monocytes and dendritic cells (Vigne et al., 2011). IL-36 agonists bind the same receptor complex and are expressed in similar cells; adding each IL-36 agonist to keratinocytes resulted in similar immunological outcomes (Swindell et al., 2018). However, certain isoforms have been described in specific disease settings such as IL-36α in arthritis (Frey et al., 2013) and IL-36γ in psoriasis (D’Erme et al., 2015), with the blocking of IL-36γ achieving a reduction in psoriasis-associated inflammation in a 3D skin model (Todorovic et al., 2019). Whether there is isoform redundancy or whether each isoform has its own role, potentially associated with location or stimulus, remains unclear.

Interleukin-36 cytokines, like all members of the IL-1 family, are expressed without a signal peptide and are not secreted via the classical secretory pathway (Rubartelli et al., 1990). Unlike IL-1 and IL-18, IL-36 cytokines do not possess a leading peptide sequence required for caspase-1 cleavage and are therefore regulated independently of the inflammasome (Barton et al., 2000). However, a role for IL-36 cytokines facilitating activation of the NLRP3 inflammasome has been described (Chi et al., 2017). IL-36 cytokines are produced as inactive, full-length proteins that must undergo N-terminal truncation 9 amino acid residues upstream of a conserved A-X-D motif for biological activity. This precise cleavage of IL-36 cytokines increased their receptor affinity over 10,000-fold (Towne et al., 2011). It is thought that IL-36 expression is regulated by epidermal growth factor receptor (EGFR) signaling (Satoh et al., 2020) and positive feedback loops associated with Th17 cytokines (Carrier et al., 2011). There has been a recent focus on proteases that can cleave and activate IL-36 cytokines. Interestingly, blocking these proteases in inflammatory disease may have therapeutic potential. The neutrophil-derived proteases elastase and proteinase 3 appear to cleave IL-36 agonist cytokines, although their non-specific protease activity rarely activated IL-36. While neutrophils are not abundantly resident at mucosal barrier surfaces, neutrophil elastase was able to specifically activate IL-36Ra (Macleod et al., 2016), suggesting neutrophils may mediate inflammation once recruited. A clear role for the cysteine protease cathepsin S activating IL-36 agonists has also been described. Crucially, cathepsin S is found in fibroblasts and keratinocytes and its activity was increased in psoriatic lesions (Ainscough et al., 2017). IL-38 lacks a signal peptide and caspase-1 cleavage site, suggesting that, unlike IL-36Ra, activation is not required. IL-38 is expressed mostly in the skin but has also been found in B-cells (Lin et al., 2001).

### The Immunological Function of the IL-36 Subfamily

Following their induction and activation, IL-36 signaling has potent effects on barrier immunity and can lead to protective responses against pathogens or drive autoinflammatory disease. IL-36 agonist signaling leads to activation of MAPK and NF-κB pathways (He et al., 2013). Downstream this results in anti-microbial peptide release from keratinocytes (Nguyen et al., 2012), increased recruitment and maturation of myeloid cells (Foster et al., 2014), increased macrophage phagocytosis and microbial killing (Tao et al., 2017), and the robust production of IL-6, IL-8, TNF, CCL3, CCL4, CCL5, CCL20, CXCL1, CXCL2, and CXCL8 (Carrier et al., 2011; Ramadas et al., 2012; Foster et al., 2014; Dietrich et al., 2016). It is likely a finely tuned balance of IL-36 agonist and antagonist activity promotes protective immunity. This is evidenced in generalized pustular psoriasis (the most severe form of psoriasis) where patients lack IL-36Ra due to a missense mutation in the IL-36Ra gene. Furthermore, IL-36Ra deficiency is associated with systemic inflammation, suggesting that uncontrolled IL-36 agonist signaling has systemic effect (Marrakchi et al., 2011).

The IL-36 subfamily also bridges innate and adaptive immunity. IL-36α/γ secreted from immune and epithelial cells directly acts on CD4^+^ T-cells and results in the release of IL-36β, which through a feedback loop promoted IL-2 secretion, T-cell expansion, and Th1 differentiation (Vigne et al., 2012). More recently, IL-36 gene expression has been associated with Th17 immunity. Here, IL-36 agonist cytokines regulated their own expression and drove the expression and function of Th17 cytokines and immunity (Carrier et al., 2011). While our knowledge of IL-36 is increasing, little information exists about the function of IL-38. The induction of IL-38 occurs in apoptotic cells to limit inflammation (Mora et al., 2016). However, as yet no induction of IL-38 in disease settings has been described apart from the inhibition of *Candida*-induced Th17 immunity in a similar manner to IL-36Ra (van de Veerdonk et al., 2012).

### Fungal Induction of the IL-36 Subfamily

Although there is good evidence describing the function of the IL-36 subfamily, there is limited evidence for the induction and function of IL-36 during fungal infection. Given the important barrier function of IL-36, these cytokines likely mediate interactions with both commensal and pathogenic fungi. *C. albicans* induced IL-36γ expression in human keratinocytes (Braegelmann et al., 2018), IL-36α/γ in TR146 cells (a human epithelial cell line), and all IL-36 agonists during an *in vivo* OPC model (Verma et al., 2018). IL-36α/γ expression was significantly increased within 24 h of OPC challenge, while IL-36β increased at 48 h. The induction of IL-36 following *Candida* challenge was dependent on candidalysin, with a candidalysin-null *Candida* strain inducing drastically reduced IL-36 expression (Verma et al., 2018). The induction of IL-36 during systemic *Candida* infection has yet to be demonstrated. However, systemic clinical infections typically arise from disrupted barrier integrity. Replicating this *in vivo* is challenging and systemic infections are achieved through intravenous injection.

The signaling events that lead to IL-36 induction following *Candida* infection have been investigated using the OPC model. Multiple signaling pathways are activated during OPC including MAPK, PI3K, and NF-κB (Moyes et al., 2010, 2014; Verma et al., 2018) and blocking p38-MAPK but not JNK-MAPK or ERK1/2 MAPK impaired IL-36α/γ expression (Verma et al., 2018). Further investigation revealed that blocking c-Fos impaired IL-36α/γ expression, reducing c-Jun increased IL-36α expression without effecting IL-36γ. In addition, when the MAPK phosphatase MKP1 (which negatively regulates p38-MAPK and JNK-MAPK) was knocked down, IL-36α/γ expression increased (Verma et al., 2018). While these results suggest p38 MAPK induces IL-36 expression and this is negatively regulated by MKP1, MAPK pathways did not fully account for IL-36 expression. Here, while NF-κB was able to mediate some IL-36α/γ expression, blocking PI3K reduced IL-36α/γ to resting levels. These data suggest significant roles for p38-MAPK, NF-KB and PI3K in inducing IL-36 expression, with PI3K playing the most prominent role (Verma et al., 2018).

*Aspergillus fumigatus* infection also resulted in IL-36 induction, interestingly in a morphology and-time dependent manner. IL-36γ was induced in human peripheral blood mononuclear cells (PBMCs) following incubation with live conidia and heat-killed hyphae, while IL-36Ra was induced following incubation with live conidia, heat-killed conidia and live hyphae. As expected, IL-36α was not induced in PBMCs (Gresnigt et al., 2013). The induction of IL-36γ during *Aspergillus* challenge was dependent on Dectin-1 Syk signaling and TLR4 (Gresnigt et al., 2013). Dectin-1 and TLR4 signaling results in NF-κB activation which, as with *Candida*, controlled IL-36α/γ expression. The CLR Dectin-2 signals through PI3K and has an important role driving Th17 immunity against *Candida* (Saijo et al., 2010; Lee et al., 2016). Aside from *Candida* and *Aspergillus*, only *Trichophyton mentagrophytes* has been shown to induce IL-36 expression (Braegelmann et al., 2018). Although not extensively investigated, the induction of IL-36 cytokines appears to be cell-type, fungal morphology and time-dependent.

### The Role of the IL-36 Subfamily in Fungal Immunology

Following their induction, the role of IL-36 cytokines during fungal infection is poorly understood. IL-36 can be solely responsible for inflammatory disease and clearly has potent immunological effects. Therefore, following fungal induction of IL-36, the cytokines likely mediate important immunological responses. In agreement with this, IL-36 is protective during mucosal *Candida* disease. IL-36R deficient mice when challenged with OPC displayed increased fungal burden and reduced IL-23 expression (Verma et al., 2018). IL-36 also induces IL-23 in macrophages isolated from psoriasis patients, suggesting a consistent link between the two cytokines (Bridgewood et al., 2018). IL-23 drives the proliferation and survival of Th17 cells vital for anti-*Candida* immunity. Mice deficient in IL-23 (IL-23p19-/-) experienced severe OPC disease associated with a lack of neutrophil recruitment and anti-microbial peptides (Conti et al., 2009). It was thought IL-1 and IL-36 worked in tandem to enhance protective Th17 immunity (Verma et al., 2017); however, IL-36R deficient mice had normal IL-17 gene expression suggesting a distinct, unconnected role for each (Verma et al., 2018). As such, the IL-36/IL-23 axis may complement the IL-1/Th17 response through an uncoupled mechanism (Verma et al., 2018). This contrasts with *Aspergillus* infection which showed blockade of IL-36R with IL-36Ra reduced IL-17 and IFN-γ responses (Gresnigt et al., 2013).

The role of IL-36 during *Candida* infection appears to be tightly linked with candidalysin activity, which was required for IL-36 induction (Verma et al., 2018). Commensal (yeast) *Candida* does not produce candidalysin and subsequently does not initiate inflammation. Instead, candidalysin is expressed when *Candida* becomes invasive (through hypha formation) and results in inflammation and the loss of barrier integrity (Moyes et al., 2016). Here, IL-36 signaling may facilitate host discrimination between commensal and pathogenic *Candida*. Furthermore, excessive IL-36 signaling is damaging and leads to inflammatory disease. Thus, *Candida* may induce IL-36 to promote inflammation, disrupt barrier integrity and enhance disease. This may potentially explain why psoriasis patients are particularly susceptible to *Candida* infection (Pietrzak et al., 2018).

The function of IL-36 during *Aspergillus* infection has been less well explored. IL-36γ is induced following *A. fumigatus* infection in a Dectin-1 Syk dependent manner. In support of this, Dectin-1 deficient mice produced defective Th17 immune responses (LeibundGut-Landmann et al., 2007) and are highly susceptible to *Aspergillus* lung infection (Werner et al., 2009). Many systemic fungal infections are acquired across mucosal surfaces where IL-36 induction has potent effects. Understanding the function of IL-36 signaling and the balance of protective and excessive responses may provide valuable therapeutic targets to mediate barrier inflammation and integrity.

### Therapeutic Potential of the IL-36 Subfamily

Modulating IL-36 signaling has been investigated with some early success. Recently, a small molecule inhibitor of IL-36γ successfully attenuated IL-36γ induced responses (Todorovic et al., 2019). A phase 1 study in generalized pustular psoriasis patients has been completed showing a monoclonal antibody against IL-36R rapidly reduced patient pustules and psoriasis severity score (Bachelez et al., 2019). Anti-IL-23 antibodies have also been trialed with success in psoriasis patients (Reich et al., 2011). While therapeutically inhibiting IL-36 signaling may produce rapid disease improvement in autoimmune settings, IL-36 has a protective role during infectious disease and a careful balance of IL-36 signaling must be achieved.

## Discussion

The IL-1 family of cytokines are central to immunity and health. It is no surprise that this family plays a crucial role during fungal infection and in determining fungal disease outcomes. Lacking IL-1 agonist activity during acute fungal disease is often severely detrimental for the immunocompromised host, resulting in fungal growth and dissemination. However, excessive IL-1 family agonist activity, either through over expression/activation or through a lack of antagonist activity, can be equally destructive. Excessive IL-1 family signaling is associated with numerous inflammatory disorders and in the context of fungal infection, can exacerbate chronic disease and lead to barrier disruption and fungal dissemination. Therefore, potent IL-1 signaling must be carefully regulated through balancing levels of protease activation and antagonist activity, to successfully promote protection and immunity.

**FIGURE 2:**
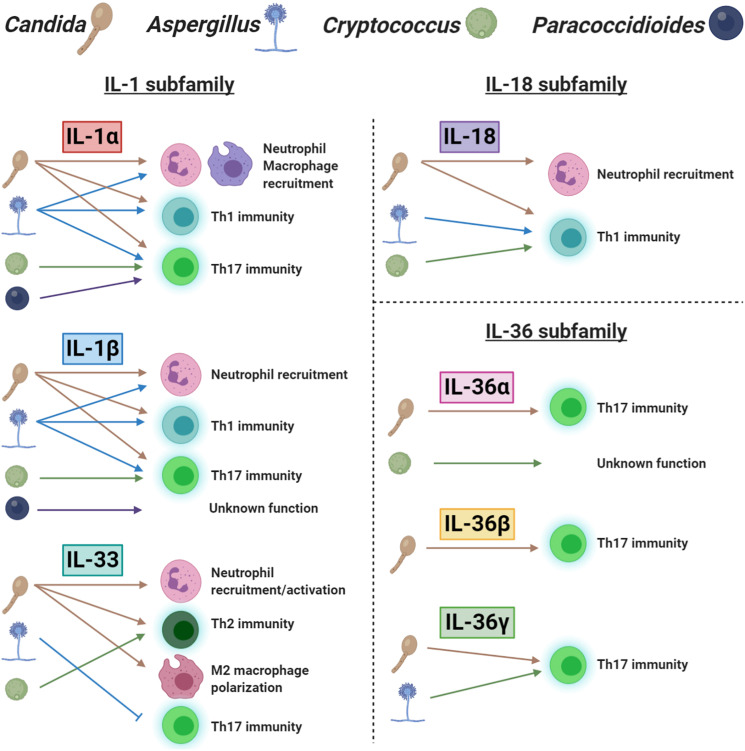
A schematic representation of the role of IL-1 family cytokines in fungal immunity. Each ligand is separated into their appropriate IL-1 subfamily. The icons for *Candida*, *Aspergillus, Cryptococcus*, and *Paracoccidioides* are labeled at the top of the figure. The presence of a fungal icon underneath a cytokine indicates induction. Arrows and blocked lines represent the functional outcome of this cytokine following induction.

Understanding of the functional role, mechanism of induction and downstream regulators of each IL-1 family member would greatly improve our knowledge of anti-fungal immunity. Here, we have reviewed the effects of different IL-1 family members that provide action at various locations and in response to multiple stimuli. We graphically summarize our current understanding of this topic in Figure 2. It is also important that our investigations of IL-1 family members consider collaboration and redundancy that occurs throughout the IL-1 family. Although individual IL-1 family members appear to have clearly defined roles in specific locations and disease settings, the interaction between IL-1 family signaling likely contributes to the overall immune response and disease outcome. While enhancing individual IL-1 family agonists or antagonists to promote disease resolution is therapeutically effective, targeting multiple members at once may provide the best outcome. Furthermore, while the exogenous administration of IL-1 family members is being investigated with some success, targeting the receptors or enzymes that drive IL-1 family induction and activation is another strategy that may provide therapeutic benefit.

As fungal diseases become an increasingly severe worldwide burden contributing to millions of deaths per year, the extensive use of immune-modulating therapies also continues to increase. Current therapies are inadequate, toxic, highly drug interactive, and frequently encounter resistance. In addition, there is no current fungal vaccine available for use. Therefore, immunotherapies that enhance anti-fungal immunity will be a vital component of future anti-fungal therapies.

## Author Contributions

JG conceptualized and wrote the manuscript. GC contributed sections and produced figures. NK and JH contributed sections. JR and JN edited the manuscript. All authors contributed to the article and approved the submitted version.

## Conflict of Interest

The authors declare that the research was conducted in the absence of any commercial or financial relationships that could be construed as a potential conflict of interest.
